# Supplementary material for: Prevalence of Mild Cognitive Impairment in the Lothian Birth Cohort 1936
Source: Alzheimer Dis Assoc Disord. 2021 Jan 20;35(3):230–6. doi: 10.1097/WAD.0000000000000433 (PMC8386587; doi:10.1097/WAD.0000000000000433)
Supplement: SUPPLEMENTARY MATERIAL [file wad-35-230-s001.docx]

Supplementary figure 1: MCI rates using normative data from the Lothian Birth Cohort 1936
